# δ-Generalized Labeled Multi-Bernoulli Filter Using Amplitude Information of Neighboring Cells

**DOI:** 10.3390/s18041153

**Published:** 2018-04-10

**Authors:** Chao Liu, Jinping Sun, Peng Lei, Yaolong Qi

**Affiliations:** School of Electronic and Information Engineering, Beihang University, Beijing 100191, China; LC2016@buaa.edu.cn (C.L.); sunjinping@buaa.edu.cn (J.S.); longgniy@163.com (Y.Q.)

**Keywords:** δ-GLMB filter, amplitude information, neighboring cells, multi-target tracking

## Abstract

The amplitude information (AI) of echoed signals plays an important role in radar target detection and tracking. A lot of research shows that the introduction of AI enables the tracking algorithm to distinguish targets from clutter better and then improves the performance of data association. The current AI-aided tracking algorithms only consider the signal amplitude in the range-azimuth cell where measurement exists. However, since radar echoes always contain backscattered signals from multiple cells, the useful information of neighboring cells would be lost if directly applying those existing methods. In order to solve this issue, a new δ-generalized labeled multi-Bernoulli (δ-GLMB) filter is proposed. It exploits the AI of radar echoes from neighboring cells to construct a united amplitude likelihood ratio, and then plugs it into the update process and the measurement-track assignment cost matrix of the δ-GLMB filter. Simulation results show that the proposed approach has better performance in target’s state and number estimation than that of the δ-GLMB only using single-cell AI in low signal-to-clutter-ratio (SCR) environment.

## 1. Introduction

The multi-target tracking (MTT) [1,2] is an important capability for modern radar data processing. Taking the pulsed radar as an example, the pulse compression and clutter suppression are first used to remove clutter and jamming signals from received echoes. Then we can extract target information via integration and detection. A threshold is usually set in detection algorithms according to the Newman-Pearson discipline, and the echo signal beyond the threshold is extracted as plots. Due to the limitations of clutter suppression or detection, these plots may originate from not only targets but also clutter. Moreover, the missed detection of targets can also occur. The plots are then put into the data preprocessing (such as plots centroid, etc.) for the preparation of target tracking. The objective of MTT is to estimate the unknown and time-varying number of targets as well as their individual states from the observation sequence. It improves the radar sensing of moving targets in current search area.

The traditional multi-target tracking paradigms, such as Joint Probabilistic Data Association (JPDA) [3] and Multiple Hypotheses Tracking (MHT) [4], implement data association followed by the Kalman filter for single target. When targets are spatially close or a large number of false alarms are present, the data association becomes very complex. Therefore, Mahler proposes the multi-target filtering theory based on Random Finite Sets (RFS) [5]. In the framework of this theory, multi-target states and sensor measurements are modeled as random finite sets, and the recursive estimation of multi-target posterior density is achieved by Bayesian multi-target filtering. The early implementations of RFS paradigm, such as probability hypothesis density (PHD) [6], cardinalized probability hypothesis density (CPHD) [7] and Multi-Bernoulli [8] filters, ignore the data association in order to reduce the computational complexity. Hence they cannot produce target tracks. On the basis, δ-generalized labeled Multi-Bernoulli (δ-GLMB) [9,10] and labeled Multi-Bernoulli (LMB) [11] filters incorporate track labels into target state and take data association as part of multi-target state estimation to realize the output of target tracks.

The traditional radar signal processing procedure is first detection and then tracking. Nevertheless, when the detection performance is unsatisfactory, the tracking accuracy will decline. In this case, the track-before-detect (TBD) strategy [12,13,14,15,16] is proposed. It gets rid of the detection in any single frame, but accumulates the likelihood ratio of continuous multiple frames. This strategy provides an effective way for detecting low observable targets. It should be noted that the tracking also carries out likelihood ratio accumulation, and the difference between them is: (1) the likelihood ratio accumulated by TBD approaches contains both dynamic states and amplitude information (AI) of the target, while the tracking approaches only contains the dynamic states; (2) TBD deals with the original echoes, and traverses all range-Doppler-azimuth cells over the observation area, while the tracking only needs to process the data after thresholding. Thus, more information amount and computational burden are involved in the TBD. This disadvantage leads to the fact that TBD is currently difficult to replace the traditional detection-and-tracking procedure in real-time radar data processing. However, it outperforms the traditional procedure in low signal-to-clutter-ratio (SCR) conditions due to the full use of amplitude difference between target and clutter. Therefore, we can see that the tracking performance in low SCR conditions could be improved by using AI.

Many works in the literature have introduced AI into tracking algorithms. In [17], the detection process of echoed signals with slow Rayleigh fluctuation in narrowband Gaussian background is analyzed. It incorporates the likelihood ratio of amplitude into the calculation of measurement-track association weights for improving performance of the tracking filter. Based on the AI aided measurement-track association (AIA-MTA) approach in [17], more studies have further been carried out. In [18], Lerro combines the AIA-MTA with the interactive multiple model (IMM), and improves the performance of track formation and maintenance in clutter environment. To solve the track initiation problem of low observable target, Cai introduces AI into the adaptive sliding window expectation-maximization algorithm combined with maximum likelihood [19]. By analytically assessing the benefits of AIA-MTA in several simple cases, Ehrman points out that the method is unsuitable for multiple targets although it is good at tracking single target in dense clutter environment. The reason is that AIA-MTA always favors the measurement with high amplitude, but ignores the measurement states [20]. Then he proposes that the normalized target amplitude likelihood function instead of the likelihood ratio should be introduced into AIA-MTA. In [21], the error probabilities of measurement-track association for Rayleigh target, fixed amplitude and Rician target are calculated. In [22,23], Ehrman further presents that the blind use of AI in the measurement-track assignment may actually reduce the association performance, because no method can be applied to all scenarios. In [24], the performance loss in heavy-tailed clutter environment is analyzed quantitatively by simulation. In [25], a modified Riccati equation is used to predict the performance of probabilistic data association filter with AI in heavy-tailed K distributed clutter. In [26,27], the amplitude likelihood ratio proposed in [17] is introduced to the PHD filter (simplified as PHD-AI). For situations of high resolution and low grazing angle, an AI assisted PHD filter in Weibull clutter background is proposed by Li [28]. In [29], Liu proposes to improve the association performance with AI in complex ground target tracking. In [30], Yuan gives an improved multi-Bernoulli filter with AI.

However, one obvious disadvantage of these existing researches is that when constructing, only the AI of the measurement cell is used for constructing the amplitude likelihood function, while that of surrounding cells is ignored. In some cases, for example, when the size of the target is large or the sampling interval of range and azimuth is small, the target echoes may occupy multiple adjacent cells. After detection process, the plots are likely to be distributed in multiple adjacent cells. This phenomenon is called spread [12] or spillover [16] of target energy. In order to reduce complexity, most of target tracking approaches require that one measurement is generated for a target in each frame [5]. Therefore, the plots centroid technology is usually applied to handle the neighboring plots, and a central measurement is obtained according to certain principles. The central measurement would be used as the only target measurement for tracking, while the other neighboring plots are discarded. If AI of surrounding cells is preserved at the step of plots centroid, the amount of available information for filtering will be greatly increased. It could help improve the tracking performance. By now, this idea has been used in some TBD approaches [12,13,16].

Based on the above analysis, this paper proposes a new δ-GLMB filter with united likelihood ratio of AI in neighboring cells in the complex Gaussian distributed clutter (simplified as GLMB-AI-UL). First, using PHD-AI [27], a δ-GLMB filter with single cell AI (simplified as GLMB-AI) is derived. Then the amplitude measurement is modeled with the point spread function employed in TBD approaches. However, different from traversing all cells in TBD to calculate the amplitude likelihood ratio, the method only considers cells around each measurement after plots centroid to obtain the likelihood ratio. Thus, its calculation cost is significantly lower than that of TBD. Finally, the likelihood ratio is introduced into δ-GLMB update process and measurement-track assignment cost matrix to improve its filtering performance.

The remainder of this paper is organized as follows. A brief overview of RFS and the δ-GLMB filter are provided in Section 2. PHD-AI is introduced in Section 3. On this basis the GLMB with united likelihood ratio of AI in neighboring resolution cells is proposed in Section 4. Section 5 presents simulation results. Conclusions are made in Section 6.

## 2. Bayesian Multi-Target Filtering

### 2.1. Basic Notations

The usage of notations in the paper follows the same way as those in [9,10]. Single-target states are denoted by small letters (e.g., x) and multi-target states are denoted by capital letters (e.g., X). In labeled RFSs, labeled target states are denoted by bold face letters (e.g., X). In addition, spaces are usually denoted by blackboard bold letters (e.g., X represents the state space, and L represents the label space). The group of all finite subsets of X is denoted by ℱ(X). A labeled single-target state x consists of a kinematic state x∈X and a label ℓ∈L. A single measurement is denoted by a small letter (e.g., z), and a set of multiple measurements is denoted by a capital letter (e.g., Z).

The standard inner product of f(x) and g(x) is defined as

〈f,g〉≜∫f(x)g(x)dx.

The multi-target exponential function is defined as

$$h^{X}≜\prod_{x\in X}h\left(x\right),$$

where h is a real-valued function, and it is usual to set $h^{⌀}=1$. The generalized Kronecker delta function is defined as

$$\delta_{Y}\left(X\right)≜\left\{ \begin{array}{l} 1,\;\mathrm{if}\;X=Y\\ 0,\;\mathrm{otherwise} \end{array}\right.,$$

where X and Y can be arbitrary arguments such as sets, vectors and integers. Meanwhile, the inclusion function is defined as

$$1_{Y}\left(X\right)≜\left\{ \begin{array}{l} 1,\;\mathrm{if}\;X\subseteq Y\\ 0,\;\mathrm{otherwise} \end{array}\right..$$

### 2.2. Bayesian Multi-Target Filtering

Assume that there exist N(k) targets $x_{k,1},\ldots ,x_{k,N\left(k\right)}$ at time k. Each of them takes values from the labeled state space X×L, and their measurements $z_{k,1},\ldots ,z_{k,M\left(k\right)}$ takes values from the observation space ℤ. As a result, the collection of targets and measurements at time k can be modeled as [9]

$$X_{k}=\left\{x_{k,1},\ldots ,x_{k,N\left(k\right)}\right\},$$

$$Z_{k}=\left\{z_{k,1},\ldots ,z_{k,M\left(k\right)}\right\}.$$

The aim of multi-target tracking is to estimate the multi-target posterior density $\pi_{0:k}\left(X_{0:k}|Z_{0:k}\right)$ based on the measurement until now. It can be realized recursively by

(1) $$\pi_{0:k}\left(X_{0:k}|Z_{0:k}\right)\propto g_{k}\left(Z_{k}|X_{k}\right)f_{k|k-1}\left(X_{k}|X_{k-1}\right)\pi_{0:k-1}\left(X_{0:k-1}|Z_{0:k-1}\right),$$

where $X_{0:k}=\left(X_{0},\ldots ,X_{k}\right)$, $Z_{0:k}=\left(Z_{0},\ldots ,Z_{k}\right)$, $g_{k}\left(\cdot |\cdot \right)$ is the multi-target likelihood function at time k, and $f_{k|k-1}\left(\cdot |\cdot \right)$ is the multi-target transition density from time k−1 to time k.

In order to obtain the multi-target posterior probability density at current moment k, we omit the dependence on past measurements for simplicity. Thus, it can be realized from time k−1 by prediction and update formulations [4,5] as follows

(2) $$\pi_{k+1|k}\left(X_{k+1}\right)=\int f_{k+1|k}(X_{k+1}|X_{k})\pi_{k}\left(X_{k}|Z_{k}\right)\delta X_{k},$$

(3) $$\pi_{k+1}\left(X_{k+1}|Z_{k+1}\right)=\frac{g_{k+1}(Z_{k+1}|X_{k+1})\pi_{k+1|k}\left(X_{k+1}\right)}{\int g_{k}(Z_{k+1}|X_{k+1})\pi_{k+1|k}\left(X_{k+1}\right)\delta X_{k+1}},$$

where the integral in Equation (2) is a set integral for any function f:ℱ(X×L)→ℝ, and defined by

(4) $$\int f(X)\delta X=\sum_{i=0}^{\infty }\frac{1}{i!}\int f(\left\{x_{1},\ldots ,x_{i}\right\})d\left(x_{1},\ldots ,x_{i}\right).$$

### 2.3. δ-GLMB Filter

Because of the paper length limit, only the update process of δ-GLMB filter is introduced herein. For more details of the filter, readers can refer to [9,10].

Let the current multi-target prediction density has the δ-GLMB as

(5) $$\pi \left(X\right)=\Delta \left(X\right)\sum_{\left(I,\xi \right)\in ℱ\left(L\right)\times \Xi }w^{\left(I,\xi \right)}\delta_{I}\left(ℒ\left(X\right)\right){\left[p^{\left(\xi \right)}\right]}^{X},$$

where $w^{\left(I,\xi \right)}$ is the weight of the prediction component (I,ξ), and $p^{\left(\xi \right)}$ is the probability density function (pdf) of a single target. Then the multi-target filtering density of current moment is also a δ-GLMB given by

(6) $$\pi \left(X|Z\right)=\Delta \left(X\right)\sum_{\left(I,\xi \right)\in ℱ\left(L\right)\times \Xi }\sum_{\theta \in \Theta \left(I\right)}w^{\left(I,\xi ,\theta \right)}\delta_{I}\left(ℒ\left(X\right)\right){\left[p^{\left(\xi ,\theta \right)}(\cdot |Z)\right]}^{X},$$

where

(7) $$w^{\left(I,\xi ,\theta \right)}\propto w^{\left(I,\xi \right)}{\left[\eta_{Z}^{\left(\xi ,\theta \right)}\right]}^{I},$$

(8) $$\eta_{Z}^{\left(\xi ,\theta \right)}\left(\ell \right)=\langle p^{\left(\xi \right)}\left(\cdot ,\ell \right),\psi_{Z}\left(\cdot ,\ell ;\theta \right)\rangle ,$$

(9) $$p^{\left(\xi ,\theta \right)}\left(x,\ell |Z\right)=\frac{p^{\left(\xi \right)}\left(x,\ell \right)\psi_{Z}\left(x,\ell ;\theta \right)}{\eta_{Z}^{\left(\xi ,\theta \right)}\left(\ell \right)},$$

(10) $$\psi_{Z}\left(x,\ell ;\theta \right)=\left\{ \begin{array}{ll} \frac{p_{D}(x,\ell )g\left(z_{\theta \left(\ell \right)}|x,\ell \right)}{k(z_{\theta (\ell )})} & \theta \left(\ell \right)>0\\ 1-p_{D}(x,\ell ) & \theta \left(\ell \right)=0 \end{array}\right.,$$

and Θ(I) denotes a subset of association maps at current time with domain I.

## 3. Amplitude Information Aided Multi-Target Filter

### 3.1. Amplitude Information Modeling

It is supposed that an augmented state including AI of the i-th target at time k is represented as

(11) $$x_{k}^{i}:=\left[{\tilde{x}}_{k}^{i};d\right],$$

where ${\tilde{x}}_{k}^{i}={\left[\begin{array}{cccc} p_{1,k}^{i} & p_{2,k}^{i} & {\dot{p}}_{1,k}^{i} & {\dot{p}}_{2,k}^{i} \end{array}\right]}^{T}$ is the basic state including positions $\left(p_{1,k}^{i},p_{2,k}^{i}\right)$ and velocities $\left({\dot{p}}_{1,k}^{i},{\dot{p}}_{2,k}^{i}\right)$, and d is the power ratio of target and clutter signals. For computational convenience, the clutter power is usually normalized, and then 1+d represents the mean normalized SCR, which is typically expressed in a logarithmic form

(12) $$SCR=10\log_{10}\left(1+d\right).$$

Each measurement contains a two-dimensional target position $\tilde{z}$ and amplitude a

(13) $$z_{k}^{i}:=\left[{\tilde{z}}_{k}^{i};a\right].$$

To simplify notations, we use x, $\tilde{x}$, z and $\tilde{z}$ to denote $x_{k}^{i}$, ${\tilde{x}}_{k}^{i}$, $z_{k}^{i}$ and ${\tilde{z}}_{k}^{i}$, respectively.

Assuming that the amplitude is independent of the dynamic state, the measurement likelihood function of the target g(z|x) and that of the clutter c(z) can be given by

(14) $$g(z|x)=g_{\tilde{z}}(\tilde{z}|x)g_{a}(a|d),\;a\geq 0,$$

(15) $$c(z)=c_{\tilde{z}}(\tilde{z})c_{a}(a),\;a\geq 0,$$

where $g_{\tilde{z}}(\tilde{z}|x)$ and $c_{\tilde{z}}(\tilde{z})$ are dynamic state likelihoods for target and clutter, respectively. $g_{a}(a|d)$ and $c_{a}(a)$ are amplitude likelihoods for target and clutter, respectively. Using these amplitude likelihoods we can compute the detection probability $p_{D}^{\tau }$ and false alarm probability $p_{FA}^{\tau }$ as

(16) $$p_{D}^{\tau }(d)=\int_{\tau }^{\infty }g_{a}(a|d)da,$$

(17) $$p_{FA}^{\tau }=\int_{\tau }^{\infty }c_{a}(a)da.$$

Then the normalized amplitude likelihood function can be expressed as

(18) $$g_{a}^{\tau }(a|d)=\frac{1}{p_{D}^{\tau }(d)}g_{a}(a|d),\;a>\tau ,$$

(19) $$c_{a}^{\tau }(a)=\frac{1}{p_{FA}^{\tau }}c_{a}(a),\;a>\tau .$$

### 3.2. Amplitude Information Aided PHD Filter

The PHD-AI filter is derived in [27], and the main results are just listed as follows. 

Let λ denote the expected number of Poisson distributed clutter before detection. Then the expected number after detection is $\lambda p_{FA}^{\tau }$, which is also Poisson distributed. The pdf of clutter after detection is given by

(20) $$f_{C}\left(K\right):=\exp\left(-\lambda p_{FA}^{\tau }\right)\prod_{z\in K}\lambda p_{FA}^{\tau }c_{a}^{\tau }(a)c_{\tilde{z}}(\tilde{z}|\tilde{x}).$$

When pdf of clutter and AI of target are incorporated, the multi-target likelihood function is modified as

(21) $$f_{k+1}\left(Z_{k+1}|X_{k+1}\right)=f_{C}\left(Z_{k+1}\right)\cdot \prod_{i}^{n}\left(1-p_{D}^{\tau }\left(d_{i}\right)\right)\times \sum_{\theta }\prod_{i:\theta \left(i\right)>0}\frac{p_{D}^{\tau }\left(d_{i}\right)g_{\tilde{z}}({\tilde{z}}_{\theta \left(i\right)}|{\tilde{x}}_{i})g_{a}^{\tau }(a_{\theta \left(i\right)}|d_{i})}{\left(1-p_{D}^{\tau }\left(d_{i}\right)\right)\lambda p_{FA}^{\tau }c_{\tilde{z}}({\tilde{z}}_{\theta \left(i\right)})c_{a}^{\tau }(a_{\theta \left(i\right)})},$$

where the summation notation represents the sum over all possible associations θ between X and Z. The difference between the above and the standard multi-object likelihood function is the introduction of amplitude likelihood ratio, i.e.,

(22) $$\Lambda^{\tau }=\frac{g_{a}^{\tau }(a_{\theta \left(i\right)}|d_{i})}{c_{a}^{\tau }(a_{\theta \left(i\right)})}.$$

Therefore the PHD pseudo-likelihood is given by

(23) $$L_{Z}\left(x\right)=\left(1-p_{D}^{\tau }\left(d\right)\right)+p_{D}^{\tau }\left(d\right)\times \sum_{z\in Z}\prod_{i:\theta \left(i\right)>0}\frac{p_{D}^{\tau }\left(d\right)g_{a}^{\tau }(a|d)g_{\tilde{z}}(\tilde{z}|\tilde{x})}{\lambda p_{FA}^{\tau }c_{\tilde{z}}(\tilde{z})c_{a}^{\tau }(a)+\langle D_{k+1|k},p_{D}^{\tau }\left(d\right)g_{\tilde{z}}\rangle }.$$

## 4. δ-GLMB Filter Using Amplitude Information of Neighboring Cells

In this section, we firstly introduce AI into the δ-GLMB filter on the basis of PHD-AI, namely the GLMB-AI. Compared with PHD-AI, an attractive advantage of GLMB-AI is the capability to output target tracks. Secondly, the point spread model used in TBD approaches is exploited to model the measurement set, and then the amplitude likelihood ratio of the neighboring cells in complex Gaussian clutter is derived. Finally, the united likelihood ratio of the neighboring cells is incorporated into the δ-GLMB update process and measurement-track assignment matrix. For simplicity, the new method is called GLMB-AI-UL.

### 4.1. GLMB-AI

#### 4.1.1. Update

According to [10], if only the dynamic state information is considered, the multi-target likelihood function is given by

(24) $$g(Z|X)=e^{-\langle k,1\rangle }k^{Z}\sum_{\theta \in \Theta \left(ℒ\left(X\right)\right)}{\left[\psi_{\tilde{z}}\left(\cdot ;\theta \right)\right]}^{X},$$

where $k=\lambda \cdot c_{\tilde{z}}(\tilde{z})$ is the intensity function of clutter, and the likelihood is

(25) $$\psi_{\tilde{z}}\left(\tilde{x},\ell ;\theta \right)=\left\{ \begin{array}{ll} \frac{p_{D}^{\tau }(\tilde{x},\ell )g\left({\tilde{z}}_{\theta \left(\ell \right)}|\tilde{x},\ell \right)}{k({\tilde{z}}_{\theta (\ell )})}, & \theta \left(\ell \right)>0\\ 1-p_{D}^{\tau }(\tilde{x},\ell ), & \theta \left(\ell \right)=0 \end{array}\right..$$

Substitute the pdf of clutter in Equation (20) into Equation (24), and the amplitude likelihood ratio in Equation (22) into Equation (25). According to the Proposition 7 in [8], we can derive the posterior pdf of GLMB-AI as

(26) $$\pi \left(X|Z\right)=\Delta \left(X\right)\sum_{\left(I,\xi \right)\in ℱ\left(L\right)\times \Xi }\sum_{\theta \in \Theta \left(I\right)}w^{\left(I,\xi ,\theta \right)}\delta_{I}\left(ℒ\left(X\right)\right){\left[p^{\left(\xi ,\theta \right)}(\cdot |Z)\right]}^{X},$$

where

(27) $$w^{\left(I,\xi ,\theta \right)}\propto w^{\left(I,\xi \right)}{\left[\eta_{Z}^{\left(\xi ,\theta \right)}\right]}^{I},$$

(28) $$\eta_{Z}^{\left(\xi ,\theta \right)}\left(\ell \right)=\langle p^{\left(\xi \right)}\left(\cdot ,\ell \right),\psi_{Z}^{\tau }\left(\cdot ,\ell ;\theta \right)\rangle ,$$

(29) $$p^{\left(\xi ,\theta \right)}\left(x,\ell |Z\right)=\frac{p^{\left(\xi \right)}\left(x,\ell \right)\psi_{Z}^{\tau }\left(x,\ell ;\theta \right)}{\eta_{Z}^{\left(\xi ,\theta \right)}\left(\ell \right)},$$

(30) $$\psi_{z}^{\tau }\left(x,\ell ;\theta \right)=\left\{ \begin{array}{ll} \frac{p_{D}^{\tau }(x,d,\ell )g_{\tilde{z}}({\tilde{z}}_{\theta \left(\ell \right)}|\tilde{x},\ell )g_{a}^{\tau }(a_{\theta \left(\ell \right)}|d,\ell )}{\lambda c_{\tilde{z}}({\tilde{z}}_{\theta \left(\ell \right)})c_{a}^{\tau }(a_{\theta \left(\ell \right)})p_{FA}^{\tau }}, & \theta \left(\ell \right)>0\\ 1-p_{D}^{\tau }(x,d,\ell ), & \theta \left(\ell \right)=0 \end{array}\right..$$

#### 4.1.2. Ranked Measurement-Track Assignment

In the update process, the ranked assignment algorithm proposed by Murty is used to calculate the assignment cost of every measurement to track and seek the least cost assignment matrices [10]. Under the condition that only the target dynamic information is considered, each assignment cost is given by

(31) $$C_{i,j}=-\ln\left(\frac{\langle p^{\left(\xi \right)}\left(\tilde{x},\ell_{i}\right),p_{D}\left(\tilde{x},\ell_{i}\right)g\left({\tilde{z}}_{j}|\tilde{x},\ell_{i}\right)\rangle }{\langle p^{\left(\xi \right)}\left(\tilde{x},\ell_{i}\right),1-p_{D}\left(\tilde{x},\ell_{i}\right)\rangle k({\tilde{z}}_{j})}\right).$$

Now we bring the target amplitude likelihood in Equation (14) and the clutter amplitude likelihood in Equation (15) into $g\left({\tilde{z}}_{j}|\tilde{x},\ell_{i}\right)$ and $k({\tilde{z}}_{j})$, and then obtain a new assignment cost as

(32) $$C_{i,j}=-\ln\left(\frac{\langle p^{\left(\xi \right)}\left(\tilde{x},\ell_{i}\right),p_{D}^{\tau }(x,d,\ell_{i})g_{\tilde{z}}({\tilde{z}}_{j}|\tilde{x},\ell_{i})g_{a}^{\tau }(a_{j}|d,\ell_{i})\rangle }{\langle p^{\left(\xi \right)}\left(\tilde{x},\ell_{i}\right),1-p_{D}\left(\tilde{x},\ell_{i}\right)\rangle k(z_{j})}\right),$$

where $k(z_{j})=\lambda p_{FA}^{\tau }c_{\tilde{z}}({\tilde{z}}_{j})c_{a}^{\tau }(a_{j})$ is the function of clutter intensity after detection. According to the representation of each single target density, the above assignment cost can be computed by Gaussian mixture or sequential Monte Carlo, and the specific calculation process can refer to [10].

### 4.2. GLMB-AI-UL

#### 4.2.1. Spread Model of Target Amplitude

**Figure 1:**
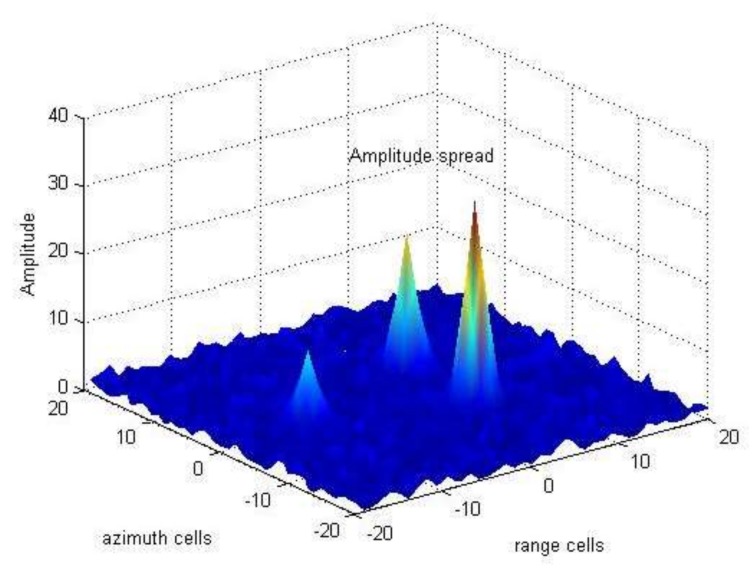
Spread phenomenon of target amplitude.

In this section, we model the amplitude spread phenomenon by taking Figure 1 as an example. In Figure 1, there are three spread targets in complex Gaussian clutter background. We use the point spread function $h^{jl}\left(x_{k}^{i}\right)$ widely used in TBD approaches to describe this phenomenon [12], i.e.,

(33) $$h^{jl}\left(x_{k}^{i}\right)=\exp\left\{-\frac{{\left(r_{k}^{j}-r_{k}^{i}\right)}^{2}}{2R}L_{r}-\frac{{\left(\theta_{k}^{l}-\theta_{k}^{i}\right)}^{2}}{2B}L_{b}\right\}$$

where $\left(r_{k}^{i},\theta_{k}^{i}\right)$ is the distance and azimuth of the sampling cell with respect to the target $x_{k}^{i}$, and $\left(r_{k}^{j},\theta_{k}^{l}\right)$, $j=1,\ldots ,N_{r}$, $l=1,\ldots ,N_{\theta }$, is the distance and azimuth of any sampling cell in the observation area. Here $N_{r}$ and $N_{\theta }$ are the total number of range bins and azimuth bins of the observation area, respectively. R and B are constants with respect to sizes of range bins and azimuth bins, and $L_{r}$ and $L_{b}$ are fading coefficients. The relationship between the target state in Equation (33) and that in Equation (11) is given by

(34) $$r_{k}^{i}=\sqrt{{\left(p_{1,k}^{i}\right)}^{2}+{\left(p_{2,k}^{i}\right)}^{2}}$$

(35) $$\theta_{k}^{i}=\arctan\left(\frac{p_{2,k}^{i}}{p_{1,k}^{i}}\right)$$

Suppose that the clutter in each unit is independent and identically distributed, and $c_{k}^{jl}$ denotes the clutter in unit (j,l) at time k. Then the measurement of this unit can be represented as

(36) $$z_{k}^{jl}=\sum_{i=1}^{N_{k}}A_{k}^{i}\exp\left(\psi_{k}^{i}\right)h^{jl}\left(x_{k}^{i}\right)+\;c_{k}^{jl}$$

where $A_{k}^{i}$ denotes amplitude measurement of the i-th target at time k, $\psi_{k}^{i}$ represents the phase of the target echo signal, and $c_{k}^{jl}$denotes zero mean complex Gaussian clutter. It should be noted that $A_{k}^{i}$ may be fixed or fluctuate according to Swerling types I~IV, and $\psi_{k}^{i}$ is usually assumed to be uniformly distributed in [0,2π). Because the target energy tends to spread only to adjacent units, let us suppose that targets are spatially so far away that each unit is only affected by the target closest to it. Then Equation (36) can be simplified to

(37) $$z_{k}^{jl}=A_{k}^{i}\exp\left(\psi_{k}^{i}\right)h^{jl}\left(x_{k}^{i}\right)+c_{k}^{jl}$$

When there is no target energy in unit (j,l), the measurement would be

(38) $$z_{k}^{jl}=c_{k}^{jl}$$

After detection, the points exceeding the threshold $\left\{z_{k}^{\left(j,l\right)}|\left|z_{k}^{\left(j,l\right)}\right|>\tau ,j=1,\ldots ,N_{r},l=1,\ldots ,N_{\theta }\right\}$ constitute the measurement set used for tracking at current scan. Herein the amplitude measurement $\left|z_{k}^{\left(j,l\right)}\right|$ is the a in Equation (13).

#### 4.2.2. Amplitude Likelihood of the Spread Unit in Complex Gaussian Background

In the situation of Swerling type I fluctuation, amplitudes of target and clutter are Rayleigh distributed. The pdf of the clutter amplitude [16] is given by

(39) $$c_{a}(a)=\frac{2a}{\sigma_{n}}\exp\left(-\frac{a^{2}}{\sigma_{n}}\right),\;a>0$$

where $\sigma_{n}$ is the covariance of the clutter amplitude. On the other hand, the pdf of the target amplitude is given by

(40) $$g_{a}(A_{k})=\frac{2A_{k}}{\sigma_{t}}\exp\left(-\frac{A_{k}{}^{2}}{\sigma_{t}}\right),\;A_{k}>0$$

where $\sigma_{t}$ is the covariance of the target amplitude. Then according to Equation (37) the amplitude of the target spread unit is

(41) $$B_{k}=\left|A_{k}\exp\left(j\varphi_{k}\right)h\right|=A_{k}h$$

By parameter transformation of Equation (40), its pdf can be obtained as follows

(42) $$g_{a}(B_{k})=\frac{2B_{k}}{h^{2}\sigma_{t}}\exp\left(-\frac{B_{k}{}^{2}}{h^{2}\sigma_{t}}\right),\;B_{k}>0$$

Therefore, the pdf of measured amplitude of target plus clutter a is the Rayleigh distribution, i.e.,

(43) $$g_{a}(a|x_{k})=\frac{2a}{\sigma_{n}+h^{2}\sigma_{t}}\exp\left(-\frac{a^{2}}{\sigma_{n}+h^{2}\sigma_{t}}\right),\;a>0$$

Let h=1 in Equation (43). We can get the amplitude pdf of the target that produces amplitude spread as

(44) $$g_{a}(a|x_{k})=\frac{2a}{\sigma_{n}+\sigma_{t}}\exp\left(-\frac{a^{2}}{\sigma_{n}+\sigma_{t}}\right),\;a>0$$

If the amplitude of one unit and its several neighboring cells is beyond the detection threshold due to the spread, the point of this unit can be extracted as the central measurement for tracking by using the points clustering. On the other hand, the AI of the neighboring cells is retained to construct the united likelihood ratio for distinguishing target from clutter. 

In Section 3.1, we use d to represent the ratio of target power to clutter power, i.e., $d=\sigma_{t}/\sigma_{n}$. To simplify the calculation, we can get $\sigma_{n}=1$ and $d=\sigma_{t}$ by normalizing the clutter power [17]. Now substituting $\sigma_{n}$ and d into Equations (39) and (44), the simplified amplitude likelihood for clutter and target respectively is represented as

(45) $$c_{a}(a)=2a\exp\left(-a^{2}\right),\;a>0$$

(46) $$g_{a}(a|d)=\frac{2a}{1+d}\exp\left(-\frac{a^{2}}{1+d}\right),\;a>0$$

And the simplified amplitude likelihood for neighboring cells is

(47) $$g_{a}(a|d,h)=\frac{2a}{1+h^{2}d}\exp\left(-\frac{a^{2}}{1+h^{2}d}\right)$$

Substituting Equation (45) into Equations (17) and (19), we obtain the probability of false alarm and the normalized amplitude likelihood for clutter as

(48a) $$p_{FA}^{\tau }=\exp\left(-\tau^{2}\right)$$

(48b) $$c_{a}^{\tau }(a)=2a\exp\left(\tau^{2}-a^{2}\right),\;a>\tau$$

where τ is the detection threshold calculated by Equation (17) based on the specified false alarm probability. Similarly, substituting Equation (46) into Equations (16) and (18), we obtain the probability of detection and the normalized amplitude likelihood for target as

(49a) $$p_{D}^{\tau }=\exp\left(-\frac{\tau^{2}}{1+d}\right)$$

(49b) $$g_{a}^{\tau }(a|d)=\frac{2a}{1+d}\exp\left(\frac{\tau^{2}-a^{2}}{1+d}\right),\;a>\tau$$

#### 4.2.3. GLMB-AI-UL

The single target measurement likelihood including amplitude likelihood in the GLMB-AI update process is given by Equation (30). Now we rewrite the amplitude likelihood in the form of united amplitude likelihood of neighboring cells. In order to distinguish it from symbols in Equation (30), the improved amplitude likelihood is expressed as

(50) $${\overset{⌣}{g}}_{a}^{\tau }(a_{\theta \left(\ell \right)}|d,\ell )={\overset{⌣}{g}}_{a}^{\tau }({\left|z_{k}^{jl}\right|}_{\theta \left(\ell \right)}|d,\ell )=g_{a}^{\tau }({\left|z_{k}^{jl}\right|}_{\theta \left(\ell \right)}|d,\ell )\times \prod_{\begin{array}{c} \left(j_{m},l_{n}\right)\in \left(N\left(j\right),M\left(l\right)\right)\\ \left(j_{m},l_{n}\right)\neq \left(j,\ell \right) \end{array}}g_{a}(\left|z_{k}^{\left(j_{m},l_{n}\right)}\right||d,\ell ,h),a_{\theta \left(\ell \right)}>\tau$$

where N(j) and M(l) are range bins and azimuth bins adjacent to the range-azimuth cell where $z_{k}^{jl}$ exists, and $\left(j_{m},l_{n}\right)$ is any range-azimuth cell affected by the amplitude spread of $z_{k}^{jl}$. Similarly, the improved amplitude likelihood of the clutter is expressed as

(51) $${\overset{⌣}{c}}_{a}^{\tau }(a_{\theta (\ell )})=c_{a}^{\tau }(a_{\theta (\ell )})\prod_{\begin{array}{c} \left(j_{m},l_{n}\right)\in \left(N\left(j\right),M\left(l\right)\right)\\ \left(j_{m},l_{n}\right)\neq \left(j,\ell \right) \end{array}}c_{a}(\left|z_{k}^{\left(j_{m},l_{n}\right)}\right|),\;a_{\theta \left(\ell \right)}>\tau$$

Substituting both Equations (50) and (51) into Equations (30) and (32), the algorithm GLMB-AI-UL can be obtained. The update operation of GLMB-AI-UL is summarized in Table 1 via pseudo code.

It should be noted that the united likelihood ratio of its nearest neighboring cells may be comparable to that of the cell with only clutter when there is a non-spread target or no target in a cell. The reason is that all the surrounding cells are clutter. In this case, the tracking performance of GLMB-AI-UL is the same as that of GLMB-AI. On the contrary, when there is a spread target, not only clutter but also the target are involved in the nearest neighboring unit. Therefore, a greater likelihood ratio would be obtained by using GLMB-AI-UL, and the association weight is improved.

## 5. Simulations

In this paper, the performance of GLMB-AI-UL is compared with that of other two algorithms, i.e., GLMB-AI and δ-GLMB (simplified as GLMB), by simulation data in three SCR scenarios. Table 2 shows the specific parameter settings.

**Figure 2:**
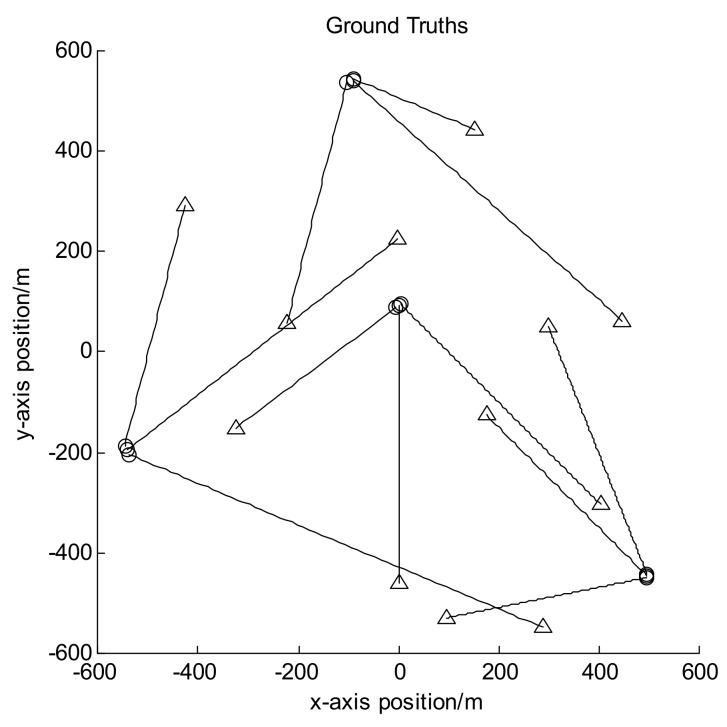
Target trajectories for scenario 1 and 2.

**Figure 3:**
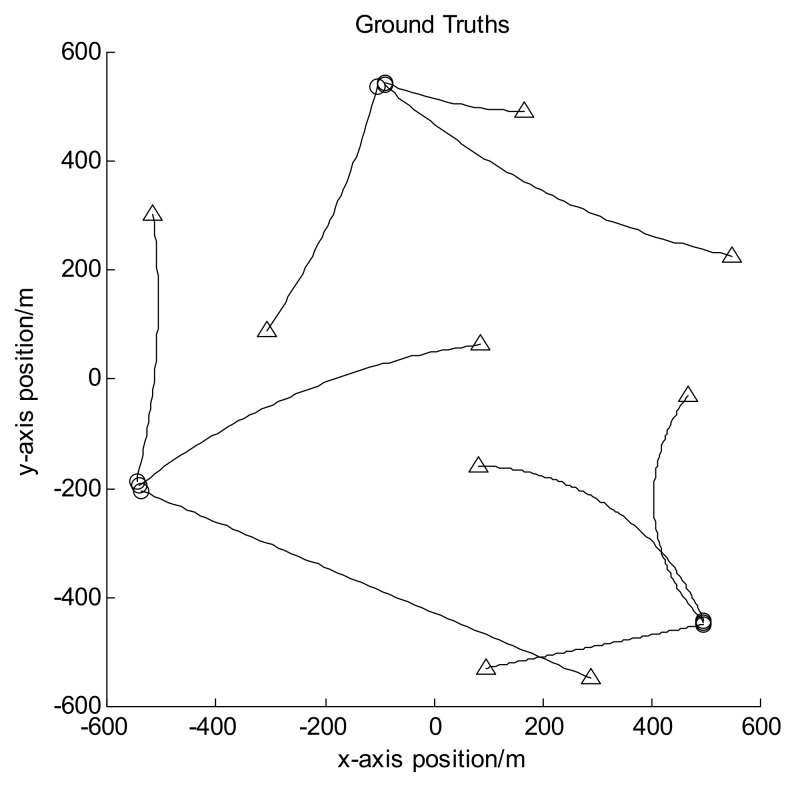
Target trajectories for scenario 3.

Let the size of observation area be [−600, 600] m × [−600, 600] m, and the observation period be 100 frames in all. In scenarios 1 and 2, there are 12 moving targets following the nearly-constant-velocity (NCV) model, and their trajectories are shown in Figure 2; while in scenario 3, there are 9 targets with some of them following the coordinated turn (CT) model, and their trajectories are shown in Figure 3. In both figures the circles denote the starting points of the target trajectories while the triangles denote corresponding endpoints, and each target has different appearance and disappearance time. Hence the target number is varying in each frame. Set the clutter number before detection to be Poisson distributed with the mean value of 800. It can be seen that after detection it is also Poisson distributed with the mean value of about 80. For GLMB without AI, the survival probability is set to be 0.9 and the detection probability to be 0.95. For GLMB-AI-UL and GLMB-AI, the survival probability is set to be 0.9, and the detection probability can be calculated by Equations (16) and (17) according to the SCR and false alarm probability. 

To evaluate the performance of GLMB-AI-UL, all the targets are simulated to spread their amplitude to neighboring cells. Due to the Rayleigh amplitude distribution, each target has different amplitude, and accordingly its spread scope is distinct as well. For the convenience of calculation, parameters of the point spread function in Equation (33) is set to be $L_{r}=L_{b}=R=B=1$. When calculating AI-UL with Equations (50) and (51), only the neighbor cells next to any central measurement are considered. The OSPA metric [31] is used to evaluate the performance of three multi-target tracking algorithms mentioned above, which is defined as follows

$${\bar{d}}_{p}^{c}\left(X,Z\right):={\left(\frac{1}{n}\left(\underset{\pi \in \prod n}{\min}\sum_{i=1}^{m}d^{\left(c\right)}{\left(x_{i},z_{i}\right)}^{p}+c^{p}\left(n-m\right)\right)\right)}^{\frac{1}{p}},$$

where we set p=1 and c=100 in simulations. In each scenario, we make 20 Monte Carlo runs for the three algorithms, and their performance are compared and analyzed by calculating the mean error.

### 5.1. Results of Scenario 1

**Figure 4:**
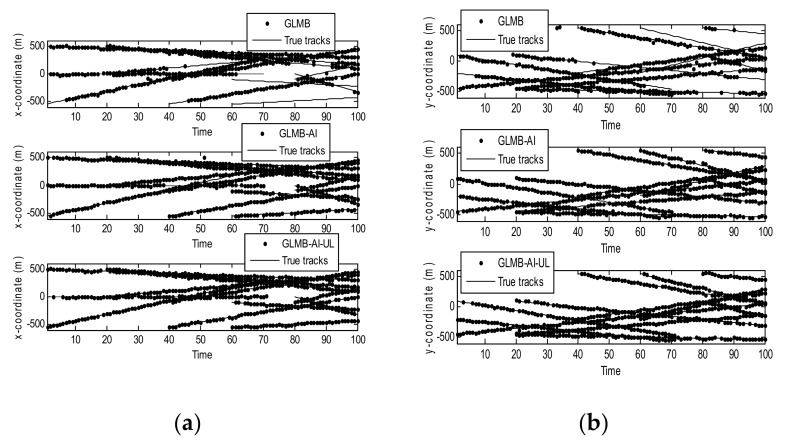
Estimates and tracks for scenario 1. (**a**) in x coordinate; (**b**) in y coordinate.

Figure 4 shows the filtering output of the three algorithms in a single operation, where Figure 4a shows the true and estimated positions in x coordinate, while Figure 4b shows the true and estimated positions in y coordinate. As can be seen that the GLMB produces some missed tracks and false tracks, while the two filters using amplitude information, i.e., the GLMB-AI and GLMB-AI-UL give much more accurate estimates. 

**Figure 5:**
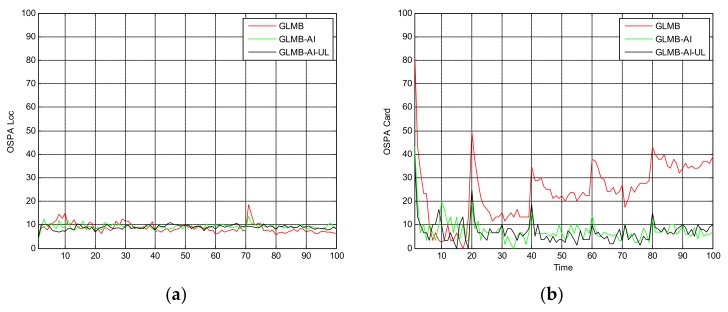
Average OSPA distance for scenario 1. (**a**) time average OSPA location distance; (**b**) time average OSPA cardinality distance.

**Figure 6:**
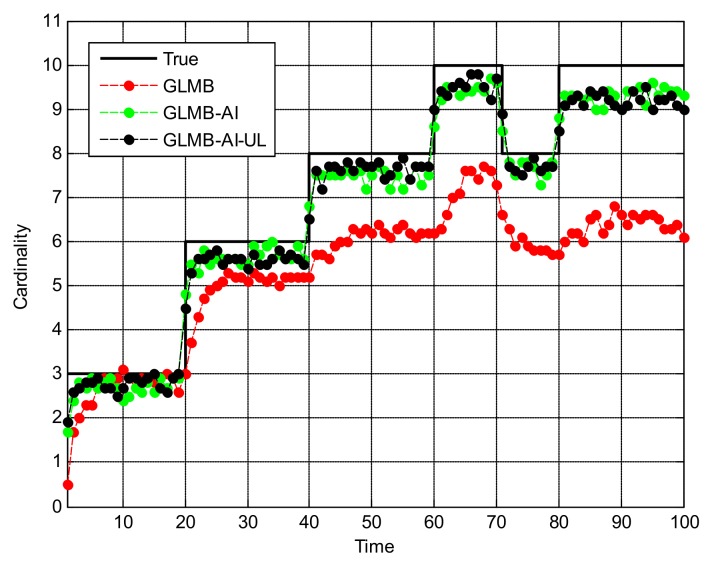
Cardinality estimates for scenario 1.

Figure 5 shows the OSPA miss distance and Figure 6 shows the comparison of cardinality estimates, and both are averaged over 20 trials. We can see from Figure 5a that the state estimation performance of GLMB-AI-UL and GLMB-AI are similar to that of the standard GLMB which only uses target dynamic information, and even at some times the GLMB performs slightly better than the GLMB-AI-UL and GLMB-AI. A possible reason for this result is that the cardinality error is ignored when calculating the location error of OSPA, which has been further discussed in [27]. GLMB may generate some false tracks due to a poorer association performance, and these tracks may be close to the actual ones of targets in space, which results in a smaller estimation error. However, in terms of the cardinality estimation performance shown in Figure 5b and Figure 6, GLMB-AI-UL and GLMB-AI are significantly better than GLMB. This is due to the exploitation of amplitude information for calculating the measurement to track association weight in the first two algorithms. In this scenario, the cardinality estimation performance of GLMB-AI-UL is almost same as that of GLMB-AI. The most possible reason for the similarity is that the SCR is high enough for GLMB-AI to distinguish target from clutter. 

### 5.2. Results of Scenario 2

**Figure 7:**
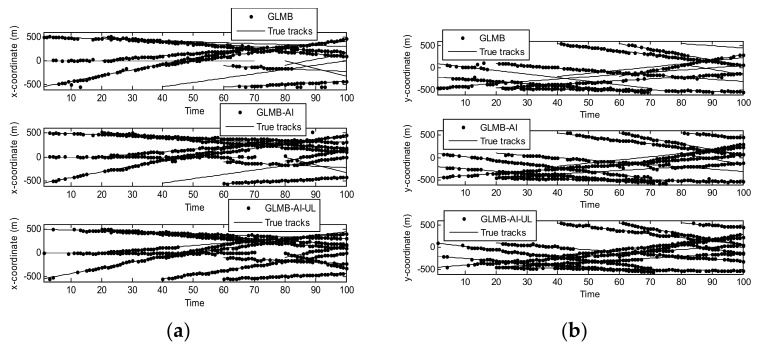
Estimates and tracks for scenario 2. (**a**) in x coordinate; (**b**) in y coordinate.

Figure 7 shows the filtering output of the three algorithms in a single run in x coordinate and y coordinate respectively. Compared with Figure 3, it can be seen that with the decrease of SCR, the state estimation performance of the three algorithms is significantly reduced. In particular, the GLMB generates more missed tracks and false tracks, and the GLMB-AI also produces some missed tracks and false tracks. Although the GLMB-AI-UL lost some plots, it still gives the most accurate state estimation, because by taking advantage of the energy of the surrounding units, a more efficient likelihood accumulation is realized.

**Figure 8:**
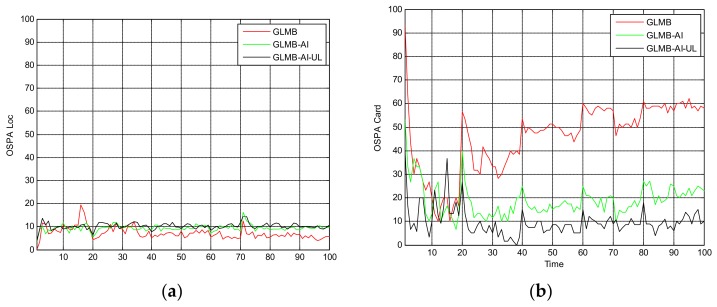
Average OSPA distance for scenario 2. (**a**) time average OSPA location distance; (**b**) time average OSPA cardinality distance.

**Figure 9:**
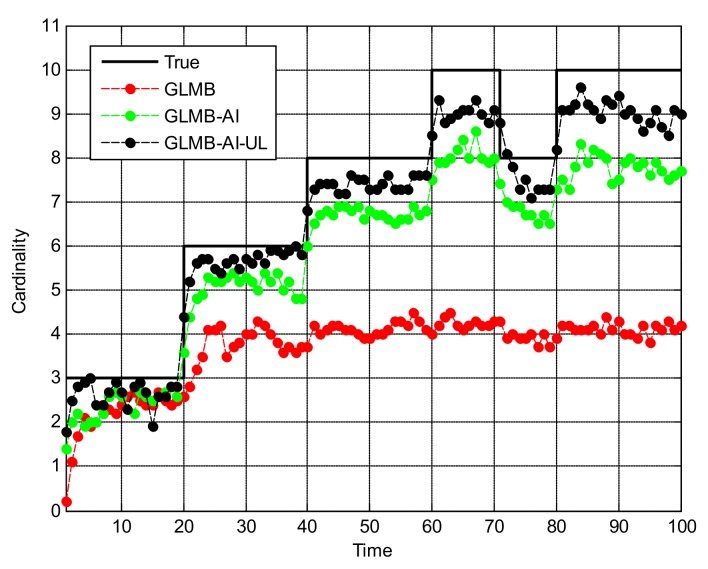
Cardinality estimates for scenario 2.

Figure 8 and Figure 9 are the comparison of averaged tracking results in scenario 2. As can be seen from Figure 8a, the state estimation performance of the three algorithms is quite similar to that of scenario 1, and the superiority of GLMB is more obvious. This is because when the SCR is lower, GLMB may produce more false tracks, which can be closer to the target. Figure 6 and Figure 8b show that the performance of cardinality estimation of GLMB-AI-UL and GLMB-AI is still significantly better than that of GLMB. However, different from scenario 1, the performance of GLMB-AI-UL is obviously better than that of GLMB-AI. The potential reason is that it is difficult to distinguish the target from clutter only by the amplitude characteristics of a single unit when the SCR is low. Nevertheless, it is possible to improve the differentiation ability by using the combined likelihood ratio of multiple resolution cells. In scenarios 1 and 2, we can also see that when the SCR is low, the deterioration degree of GLMB is the largest, and followed by GLMB-AI, which uses only single unit amplitude information, whereas GLMB-AI-UL with AI of multiple units is the most robust to strong clutter.

### 5.3. Results of Scenario 3

**Figure 10:**
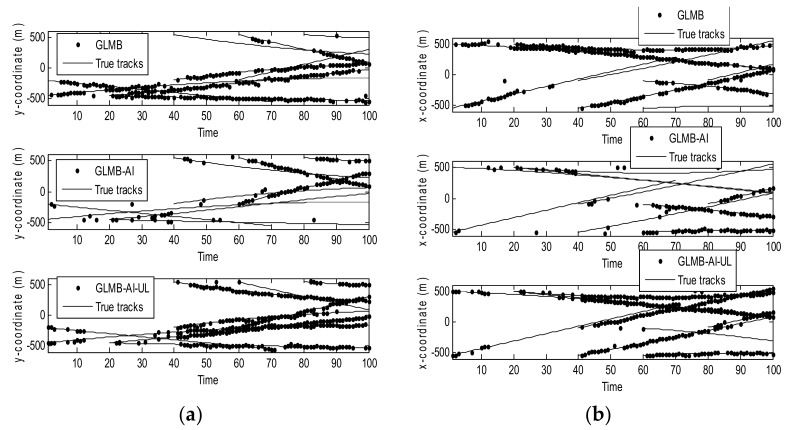
Estimates and tracks for scenario 3. (**a**) in x coordinate; (**b**) in y coordinate.

**Figure 11:**
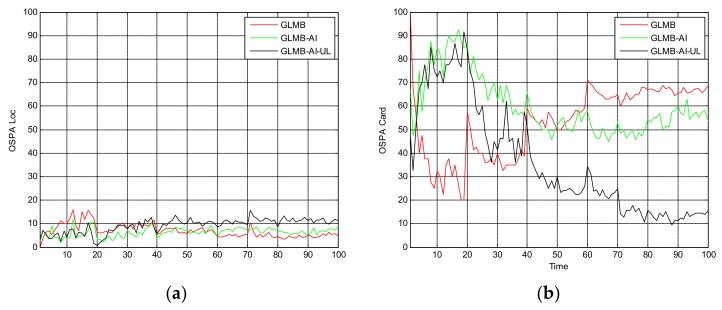
Average OSPA distance for scenario 3. (**a**) time average OSPA location distance; (**b**) time average OSPA cardinality distance.

**Figure 12:**
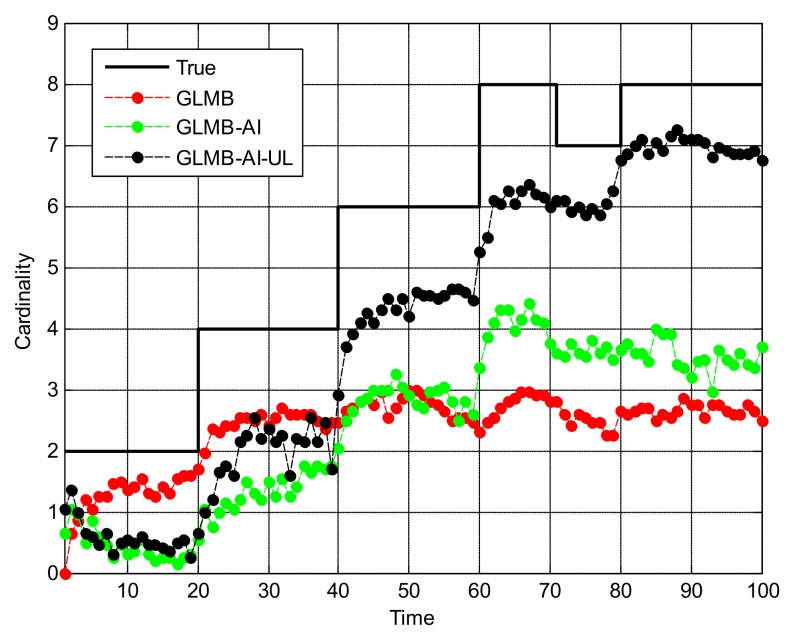
Cardinality estimates for scenario 3.

Figure 10, Figure 11 and Figure 12 are the comparison of tracking results in scenario 3. Unlike the previous two scenarios, the motion of targets in this scenario is no longer linear, but rather maneuvering, and the SCR is set with a very low value of 8 dB. 

From Figure 10 we can see that in this scenario, the tracking performance of the three algorithms is significantly reduced, and many missed tracks and false tracks are generated, but the GLMB-AI-UL still outperforms the other two algorithms, which proves the validity of the proposed method once again. From Figure 11b and Figure 12 we can see that except for the initial 40 frames, the cardinality estimation performance of the GLMB-AI-UL is always the best in the rest of the filtering time. 

Table 3 is the average running time of each algorithm in the three scenarios. As can be seen from this table, in scenario 1, when the SCR is relatively high, the filtering performance of GLMB is good, and its calculation speed is the highest. This is because the calculation of amplitude information results in an increase in computational cost. However, when the SCR was reduced, in scenario 2, the GLMB’s running time is only slightly reduced, but the elapsed time of the latter two algorithms with amplitude information is greatly reduced. This is because as the SCR decreases, the number of target-generated measurements decreases, and the utilization of amplitude information enhances the computational efficiency of the measurement-track assignment matrix. When the SCR is further reduced, the running time of the proposed GLMB-AI-UL continues decreasing, but that of the GLMB and GLMB-AI increase a lot, and the GLMB-AI-UL outperforms the other two algorithms in terms of computational cost. A reasonable explanation for this phenomenon is that, in this scenario, the SCR is such low that only few target-generated measurements exceed the threshold. This will reduce the computational cost on one hand, but on the other hand, the low SCR leads to a poorer computational efficiency of the measurement-track assignment matrix for the GLMB and GLMB-AI. Especially, the exploitation of amplitude information only of a single unit is less effective, which results in a poor performance for GLMB-AI, even worse than that of GLMB without using amplitude information. However, using the amplitude information of multiple adjacent units can evidently improve the performance. Thus, the proposed method is of great value in practical radar applications, especially when the SCR is very low.

The simulations of this paper are carried out with Matlab, and the proposed algorithm seems time consuming. However, if the procedure is written in C language, the running time will be greatly reduced. Therefore, this algorithm is suitable for real-time radar applications, particularly for low SCR environments.

## 6. Conclusions

This paper proposes a new δ-GLMB filter using the united amplitude likelihood ratio of neighboring cells. It can deal with radar target echoes from multiple range-azimuth cells in the background of complex Gaussian clutter. The filter needs to retain the amplitude information of a few cells around the central measurement in the plots centroid step, and then the information is exploited to construct a united likelihood ratio. By combining this likelihood ratio into the δ-GLMB update process and measurement-track assignment cost matrix, the ability of δ-GLMB to distinguish targets from clutter can be improved. Compared with δ-GLMB using amplitude information of single cells, the proposed algorithm can obtain more accurate target’s state and number estimation in low SCR environment. It is known that δ-GLMB not only usually has better tracking performance than PHD and Multi-Bernoulli filters, but also outputs target tracks. Therefore, compared with existing PHD and Multi-Bernoulli filters with the amplitude information of single cells, the proposed algorithm could obtain more accurate target’s state and number estimation as well as the output of target tracks. 

## Figures and Tables

**Table 1:** Update of the GLMB-AI-UL.

**input:** ${\left\{\left(I^{\left(h\right)},\xi^{\left(h\right)},w^{\left(h\right)},p^{\left(h\right)},T^{\left(h\right)}\right)\right\}}_{h=1}^{H},Z$ **output:** ${\left\{\left(I^{\left(h,j\right)},\xi^{\left(h,j\right)},w^{\left(h,j\right)},p^{\left(h,j\right)}\right)\right\}}_{\left(h,j\right)=\left(1,1\right)}^{\left(H,T^{\left(h\right)}\right)}$
for h=1:H $C_{Z}^{\left(h\right)}:=C_{Z}^{\left(I^{\left(h\right)},\xi^{\left(h\right)}\right)}$according to Equations (32), (50) and (51) ${\left\{\theta^{\left(h,j\right)}\right\}}_{j=1}^{T^{\left(h\right)}}:=$ranked assignment $\left(Z,I^{\left(h\right)},C_{Z}^{\left(h\right)},T^{\left(h\right)}\right)$ for $j=1:T^{\left(h\right)}$ $\eta_{Z}^{\left(h,j\right)}:=\eta_{Z}^{\left(\xi^{\left(h\right)},\theta^{\left(h,j\right)}\right)}$ according to Equation (28) $p^{\left(h,j\right)}:=p^{\left(\xi^{\left(h\right)},\theta^{\left(h,j\right)}\right)}\left(\cdot |Z\right)$ according to Equation (29) $w^{\left(h,j\right)}:=w^{\left(h\right)}{\left[\eta_{Z}^{\left(h,j\right)}\right]}^{I^{\left(h\right)}}$ $I^{\left(h,j\right)}:=I^{\left(h\right)}$ $\xi^{\left(h,j\right)}:=\left(\xi^{\left(h\right)},\theta^{\left(h,j\right)}\right)$ end end normalize weights ${\left\{w^{\left(h,j\right)}\right\}}_{\left(h,j\right)=\left(1,1\right)}^{\left(H,T^{\left(h\right)}\right)}$

**Table 2:** Scenario parameters.

Scenario	SCR	Mean Number of Clutter Before Detection λ	False Alarm Probability P_fa_
1	15 dB	800	0.1
2	10 dB	800	0.1
3	8 dB	800	0.1

**Table 3:** Running time averaged over 20 trials.

Scenario	GLMB	GLMB-AI	GLMB-AI-UL
1	300.6724 s	403.6110 s	433.2928 s
2	293.0126 s	343.4141 s	355.5296 s
3	340.1094 s	379.4045 s	333.7122 s
